# Construction of a dense genetic map of the *Malus fusca* fire blight resistant accession MAL0045 using tunable genotyping-by-sequencing SNPs and microsatellites

**DOI:** 10.1038/s41598-020-73393-6

**Published:** 2020-10-01

**Authors:** Ofere Francis Emeriewen, Klaus Richter, Thomas Berner, Jens Keilwagen, Patrick S. Schnable, Mickael Malnoy, Andreas Peil

**Affiliations:** 1 Julius Kühn Institute (JKI) – Federal Research Centre for Cultivated Plants, Institute for Breeding Research on Fruit Crops, Pillnitzer Platz 3a, 01326 Dresden, Germany; 2 Julius Kühn Institute (JKI) – Federal Research Centre for Cultivated Plants, Institute for Resistance Research and Stress Tolerance, Erwin-Baur-Str. 27, 06484 Quedlinburg, Germany; 3 Julius Kühn Institute (JKI) – Federal Research Centre for Cultivated Plants, Institute for Biosafety in Plant Biotechnology, Erwin-Baur-Str. 27, 06484 Quedlinburg, Germany; 4 Data2Bio LLC, Ames, IA 50011-3650 USA; 5 Plant Sciences Institute, Iowa State University, 2035B Carver, Ames, IA 50011-3650 USA; 6 Research and Innovation Centre, Genomics and Biology of Fruit Crops Department, Fondazione Edmund Mach, Via E. Mach, 1, 38010 San Michele all ‘Adige (Trentino), Italy

**Keywords:** Genetics, Plant sciences

## Abstract

Although, the Pacific crabapple, *Malus fusca*, is a hardy and disease resistant species, studies relating to the genetics of its unique traits are very limited partly due to the lack of a genetic map of this interesting wild apple. An accession of *M. fusca* (MAL0045) of Julius Kühn-Institut collection in Germany is highly resistant to fire blight disease, incited by different strains of the causative pathogen—*Erwinia amylovora*. This is the most destructive bacterial disease of *Malus* of which most of the domesticated apples (*Malus domestica*) are susceptible. Using a scarcely dense genetic map derived from a population of 134 individuals of MAL0045 × ‘Idared’, the locus (*Mfu10*) controlling fire blight resistance mapped on linkage group 10 (LG10) and explained up to 66% of the phenotypic variance with different strains. Although the development of robust and tightly linked molecular markers on LG10 through chromosome walking approach led to the identification of a major candidate gene, any minor effect locus remained elusive possibly due to the lack of marker density of the entire genetic map. Therefore, we have developed a dense genetic map of *M. fusca* using tunable genotyping-by-sequencing (tGBS) approach. Of thousands of de novo SNPs identified, 2677 were informative in *M. fusca* and 90.5% of these successfully mapped. In addition, integration of SNP data and microsatellite (SSR) data resulted in a final map comprising 17 LGs with 613 loci spanning 1081.35 centi Morgan (cM). This map will serve as a template for mapping using different strains of the pathogen.

## Introduction

The genus *Malus* Mill. is classed under the Rosaceae family of flowering plants and comprise wild apples and their domesticated relative. The wild apple species *Malus fusca* (Raf.) C.K. Schneid., also called Pacific or Oregon crabapple, is a small deciduous tree that naturally occurs on mesic habitats along the pacific coast of North America, specifically from the northern part of California to the Kenai Peninsula in Alaska^1^. Based on AFLP analyses^2^ and nuclear ribosomal and chloroplast DNA^3^, the Pacific crabapple was categorized within the group of species that are native to central Asia. *M. fusca* is a very hardy and disease resistant species, which tolerates moist soils better than other crabapples and severe frosts up to – 57 °C^4^.

The domesticated apple (*Malus domestica* Borkh.), is an important fruit crop consumed worldwide and one of the most studied members of the Rosaceae family. It is believed that the progenitors of apple are the wild apple of Central Asia and its close relatives, with Kazakhstan being a critical location in apple domestication^5,6^. Seed dispersal from Central Asia through to West Europe following human migration through trade routes allowed for hybridization and introgression with wild apple species such as the Siberian crabapple (*M*. *baccata*), Caucasian crabapple (*M*. *orientalis*) and the European crabapple (*M*. *sylvestris*)^7^, important factors leading to the domestication of apple. Thus, the domesticated apple is a complex hybrid with genetic and phenotypic variations across cultivars^8^. Apple consists of x = 17 chromosomes, and although most apple genotypes are diploid (2n = 34), other ploidy levels have been identified in *Malus*^9^. The use of structural and functional genomics vis-à-vis mapping of genetic markers and genes to specific chromosomes, as well as the sequencing of the apple genomes has increased our understanding of the domesticated apple and identified *M. sieversii* as the main progenitor of *M. domestica*^10,11^. An approximate sequence size of 643 Mb was reported for the reference cultivar ‘Golden Delicious’^11^ and around 651 Mb for the doubled haploid genotype GDDH13^10^.

Since the first sets of apple genetic linkage maps based on isozymes, RAPDs (Randomly Amplified Polymorphic DNA) and AFLPs (Amplified Fragment Length Polymorphism) were published^12–14^, many other linkage maps have now been established from bi-parental crosses of cultivars, crabapples and rootstocks, and for several traits, using robust and reproducible DNA marker technologies such as simple sequence repeat (SSR or microsatellites) markers and single nucleotide polymorphism (SNP) markers (e.g.^15–22^). Genetic linkage maps of apple are accessible at https://www.rosaceae.org.

The apple genome has an abundance of highly polymorphic and co-dominant microsatellites^18,23^. This PCR-based marker approach is suitable for genotyping of large populations due to their easy transferability and ability for multiplexing several in a single PCR reaction. Numerous SSRs were developed and mapped and form the core of published genetic maps in apple^15,17,19–21,23^. However, SSRs require huge investment of time for their development and mapping^20^. In the last decade, a generic, hybridization-based fingerprinting method culminated in the development of DArT (Diversity Arrays Technology) SNP markers^24^. DArTs, first applied in rice, were applied successfully in other plant species including members of the Rosaceae^25–28^.

Genome-wide SNP identification in *Malus* has been achieved with whole genome genotyping (WGG) array. Thus, thousands of SNP markers have been developed following re-sequencing data derived from 27 apple cultivars from the International RosBREED SNP Consortium (IRSC)^29^ as well as from the genomes of 13 apple cultivars and a crabapple species (*M*. *micromalus*)^30^. An 480 k Axiom apple array was developed after high-depth resequencing of 63 cultivars representing most of the genetic diversity in cultivated apple^31^. Furthermore, a high-density SNP integrated genetic linkage map (iGLMap) was developed in apple and includes a large number of markers, families and individuals^32^. Although in recent times costs for high-throughput next-generation sequencing (NGS) have reduced tremendously, the application of NGS to genetic mapping in apple is still insufficient^33,34^. Nevertheless, genotyping-by-sequencing (GBS^35^) has gradually become prominent. GBS, a targeted sequencing technique based on reducing genome complexity with restriction enzymes, is a cost-effective method that allows for robust and reliable de novo SNP identification^35–37^ and has now been applied in some members of Rosaceae including apple and pear^33,34,38^. Although conventional GBS (cGBS) allows for robust SNP identification, major limitations of this technique are high rates of missing data and genotyping errors^39^. Tunable GBS (tGBS) addresses some of these limitations by employing two restriction enzymes to generate overhangs in opposite orientations to which single-strand oligos rather than double-strand adaptors are ligated, thereby ensuring that only double-digested fragments are amplified and sequenced^39^. Unlike cGBS, during polymerase chain reaction (PCR) amplification in tGBS, selective nucleotides at the 3′-end of PCR primers achieve additional genome reduction.

Dense genetic linkage maps are critical for molecular breeding, identifying quantitative trait loci and genes controlling important agronomic traits in plant species. DArT and *M*. *domestica* SNPs^11^ markers applied to *M*. *fusca* accession MAL0045 × ‘Idared’ population provided insights in to a specific genetic locus controlling fire blight resistance in this wild apple accession, with DArT markers significantly linked with resistance^25^. Since QTL mapping relies on the correct ordering of markers along any chromosome^32^, already published apple SSR markers and SSRs developed from the ‘Golden Delicious’ sequences^11^ were applied to the *M*. *fusca* × ‘Idared’ population as anchor markers to develop a draft genetic map and to precisely determine the position of the fire blight resistance locus of *M*. *fusca* mapped on linkage group 10 (Mfu10)^16,40^. Only 1% of ‘Golden Delicious’ SNPs^11^ were transferable to *M. fusca* and although DArT and SSR markers were applied, the draft map remained scarcely dense with a few linkage groups of apple reference maps, still missing^16^. Thus, it was imperative to construct a dense map of this fire blight resistant accession, which would be comparable to reference *Malus* maps, and serve as template for QTL mapping with different strains of *Erwinia amylovora*—the causal pathogen of fire blight. Another rationale for developing a dense and complete map of *M. fusca* was to investigate if minor QTLs contributing to resistance to fire blight could be detected. Here, we report the use of tunable genotyping-by-sequencing (tGBS) markers to facilitate the construction of a dense genetic linkage map of *M*. *fusca* and the subsequent merging with the initial sets of SSR marker data^25^. Using previous data for three strains of *E. amylovora*^41,42^ and a new strain together with the constructed tGBS map as template, all previously detected fire blight resistance QTLs were confirmed. However, no minor locus was detected with all the aforementioned strains.

## Material and methods

### Plant materials

Progenies of crosses between *M*. *fusca* (acc. No. MAL0045) and ‘Idared’, and both parents were investigated. The progenies were composed of 116 individuals from the cross MAL0045 × ‘Idared’ (population number 05210^25^) established in 2005, 45 genotypes resulting from a cross ‘Idared’ × MAL0045 (population number 09260) done in 2008, and 21 selected recombinants from the same population planted in 2012 (population number 12229, planted for the identification of recombinants for the fire blight resistance QTL on LG10^40^ of *M. fusca*)*.* Additionally, four more *M. fusca* accessions were used. All genotypes are being maintained in the experimental fields of Julius Kühn-Institut, Dresden-Pillnitz, Germany. Young leaves of the material described above including three replicates of each parent were harvested from trees, lyophilized and sent to Data2Bio, LLC (Ames, Iowa, USA) for DNA extraction and tGBS analyses.

### tGBS SNP-type genotyping

Samples were sequenced using five runs on an Ion Proton Instrument by Data2Bio LLC, (Ames, Iowa, USA).

#### Trimming of sequencing reads

Prior to read mapping, raw reads were preprocessed with the trimming software Lucy^43,44^ according to default parameters. Thereby the nucleotides of each raw read was scanned for low quality regions and bases with PHRED quality value < 15, i.e., error rates of at least 3%, were removed^45,46^. In addition, the remaining nucleotides were scanned using overlapping windows of 10 bp and sequences beyond the last window with an average quality value less than the specified threshold were truncated.

#### Read mapping to reference genome assembly

The reference genome *Malus domestica* Whole Genome v3.0.a1 Assembly was downloaded from https://www.rosaceae.org/species/malus/malus_x_domestica/genome_v3.0.a1. Trimmed reads were mapped to the reference genome assembly using GSNAP^47^. A confidently mapped read (≤ 2 mismatches every 36 bp and less than five bases for every 75 bp as tails) was retained for subsequent analyses only if it mapped uniquely in the genome.

#### SNP calling

These uniquely mapped reads were used for SNP discovery. Potential SNP sites were carefully examined and putative homozygous and heterozygous SNPs were identified in each sample separately using different criteria for homozygous and heterozygous SNP calling, respectively. Data2Bio has previously generated custom scripts for SNP calling bioinformatics pipeline available at https://schnablelab.plantgenomics.iastate.edu/software/123SNP/^48^, although the pipeline has evolved over time. Thus:

- A SNP site was classified as homozygous in a given sample if at least five reads supported the major common allele at that site and at least 90% of all mapped reads covering that site shared the same nucleotide.
- A SNP was classified as heterozygous in a given sample if at least one read supported each of at least two different alleles and each of the two alleles comprised more than 20% of the reads mapped to that site. In addition, it was required that the sum of the number of reads supporting those two alleles were at least equal to five and comprised at least 90% of all reads covering that site.

Furthermore, the generated SNPs were filtered to define a specific minimum call rate of 70% across all samples (MCR70). Additional filtering criteria were: allele number = 2; number of genotypes ≥ 2; 10 ≤ minor allele frequency (MAF) ≤ 40%; and 35% ≤ heterozygosity rate ≤ 65%. Similar to Li et al.^49^ and Zheng et al.^50^, these tGBS parameters were established empirically with a focus on minimizing false positive and false negative SNP calls.

In addition, SNPs identified in the other four wild *Malus fusca* samples (MAL0200, MAL0289, MAL0357, and MAL0768) and the two parent samples (MAL0045 and ‘Idared’) were used to build a phylogenetic tree. SNP calling was performed using uniquely mapped reads from the six samples independently. A phylogenetic tree was built with these SNPs using the maximum likelihood based PhyML3.0^51^.

### Construction of genetic linkage maps

The *M*. *fusca* tGBS SNP genetic map was constructed with software package R/qtl at an LOD of 18^52^ using MRC70 SNPs described initially with only individuals of populations 05210 and 09260.

For the integration of the SSR data established on the 05210 population^16^ to the tGBS SNP map, individuals with missing SSR data were genotyped with such SSR markers. Furthermore, individuals of the 09260 population that were included in the tGBS analyses possessed no original SSR data and hence were genotyped with 53 SSR markers already found to be polymorphic in the 05210 population^16^. Genotyping was performed by multiplex PCR with up to 5 SSR markers using the Type-It Kit (Qiagen, Hilden, Germany) with the following conditions: 95 °C for 5 min, followed by 30 cycles of 95 °C for 1 min, 60 °C for 1 min 30 s, and 72 °C for 30 s, and a final extension for 30 min at 60 °C. PCR fragments were then analysed either on a CEQ 2000XL DNA Sequencer (Beckman Coulter, Germany) or on a 3500/3500xL Genetic Analyzer (Applied Biosystems, Germany). Sample preparations for both systems of fragment analyses are as previously reported in Emeriewen et al.^16^.

For mapping, SNP markers showing identical genotype profiles were reduced to one and markers expressing unreliable double recombinants were removed from analysis. Mapping was performed using JoinMap 4.0^53^ at an LOD threshold of 12.0–18.0. Linkage groups were assigned by comparing results with the location of SSR markers in the reference genetic linkage maps of apple.

### Mapping of markers informative in *M. fusca* to physical chromosomes of *M. domestica*

Positions of SNP markers informative in *M. fusca* on physical *M. domestica* chromosomes were determined by blasting the merged reads containing the respective SNPs (sequence length from 220 to 401 bp, Table S1) to the Golden Delicious Doubled Haploid genome (GDDH13). Only the best hits were taken into account. The position of SSRs on GDDH13 was determined by blasting both forward and reverse primers to GDDH13. If the distance of both primers was reasonable, the SSR could be placed on GDDH13, otherwise the SSR was discarded. These positions of the SSRs were used for the alignment of the genetic linkage map to the physical map.

### Phenotypic evaluation and QTL-mapping

Artificial shoot inoculation of individuals with *E. amylovora* strains Ea222, Ea3049, Ea1189, and ZYRKD3-1 (an AvrRpt2_EA_ effector deletion mutant of Ea1189), as well as disease evaluation was performed as described by Peil, et al.^54^. Briefly, up to 10 replicates of each individual were inoculated with each strain, and 28 days post inoculation (dpi), fire blight lesion length (cm) was measured and converted to percent lesion length (PLL) by dividing the necrotic shoot by the total shoot length and multiplying by 100. Phenotypic data for percent lesion length (PLL) of 05210 individuals for strains Ea222 (data from^16^), Ea3049 (data from Emeriewen et al.^41^) and ZYRKD3-1 (data from Emeriewen et al.^42^), were complemented by data obtained from inoculation of 05210 and 09260 individuals with Ea1189, and inoculation of 09260 individuals with Ea222, Ea3049, and ZYRKD3-1. Phenotypic data of individuals for each strain as well as the average of all strains and the created map as template were used for QTL-mapping, which was performed with MapQTL5^55^.

## Results

### tGBS SNP identification

From 188 samples submitted to Data2Bio, 460,552,947 Quality Trimmed tGBS reads, with an average length of 127 base pairs (bp), were generated, and 46.4% of these could be uniquely aligned (i.e., aligned to a single location) to the ‘Golden Delicious’ reference genome. Reads that aligned to more than one location were excluded in subsequent analyses. From uniquely aligned reads, 696,798 polymorphic sites were identified. SNP filtering led to the identification of 39,496 SNPs. Excessive rates of heterozygous SNPs were observed for 9 individuals which were excluded from subsequent analyses, involving 154 genotypes of populations 05210 and 09260 and 19 recombinants. Subsequently, further filtering of SNP set led to the identification of a subset of 11,998 SNPs (Table S1) called in at least 70% of samples (described as MCR70 SNPs in methods). Figure S1 shows the mapping population SNP classification.

### Genetic map of *M*. *fusca* based on tGBS SNPs

Genetic mapping was performed with only individuals of populations 05210 and 09260. SNPs found to be informative in *M. fusca* were 2677 of which 109 SNPs showing distorted segregation were discarded. Six individuals were removed from analysis due to an excessive number of cross overs, i.e., in the end 148 progeny were used for map construction. In total 2424 SNPs, representing 90.5% were successfully mapped. The resulting genetic map (Fig. S2) consists of 17 linkage groups (LGs) with a minimum SNP number of 94, the highest with 205 SNPs and an overall genetic length of 2560.8 cM (Table 1).

**Table 1:** tGBS SNPs mapped across 17 linkage groups of *M*. fusca and 17 linkage groups representing the *M. fusca* integrated map.

Corresponding apple chromosome	*M. fusca* tGBS SNP map			*M. fusca* integrated map			
	LG	Number of SNPs	Length (cM)	LG	Number of markers	Length (cM)	Largest gap (cM)
Chr1	16	94	135.7	1	41	73.56	9.98
Chr2	10	134	171.6	2	47	69.80	7.60
Chr3	9	143	115.5	3	52	73.99	4.20
Chr4	17	94	176.6	4	37	60.81	7.35
Chr5	1	205	203.6	5	39	70.62	7.80
Chr6	15	98	107.5	6	30	55.21	6.49
Chr7	7	152	187.4	7	34	70.47	17.98
Chr8	13	118	121.0	8	33	64.74	8.65
Chr9	6	154	122.5	9	33	49.27	4.28
Chr10	4*	182	159.9	10	37	69.35	8.05
Chr11	2	191	218.2	11	34	72.82	9.13
Chr12	5	155	167.2	12	34	62.04	7.16
Chr13	12	134	105.2	13	33	69.45	10.39
Chr14	14	98	146.2	14	25	34.68	4.18
Chr15	8	149	180.7	15	31	73.54	19.79
Chr16	11	137	107.6	16	27	53.44	14.49
Chr17	3	186	134.4	17	46	57.56	4.98
Overall	17	2,424	2,560.8	17	613	1081.35	

*LG* linkage group.

*LG in the tGBS SNP map where *Mfu10* is located.

### Linkage mapping to integrate tGBS and microsatellite data

Prior to mapping with all marker data (i.e. tGBS SNP and SSRs), redundant SNPs as well as SNPs raising questionable double recombination were discarded. Redundancy in this regard was defined as SNPs clustering together with same segregating pattern. In such cases, only one SNP was chosen. These led to the exclusion of 1864 of 2424 SNPs.

**Figure 1:**
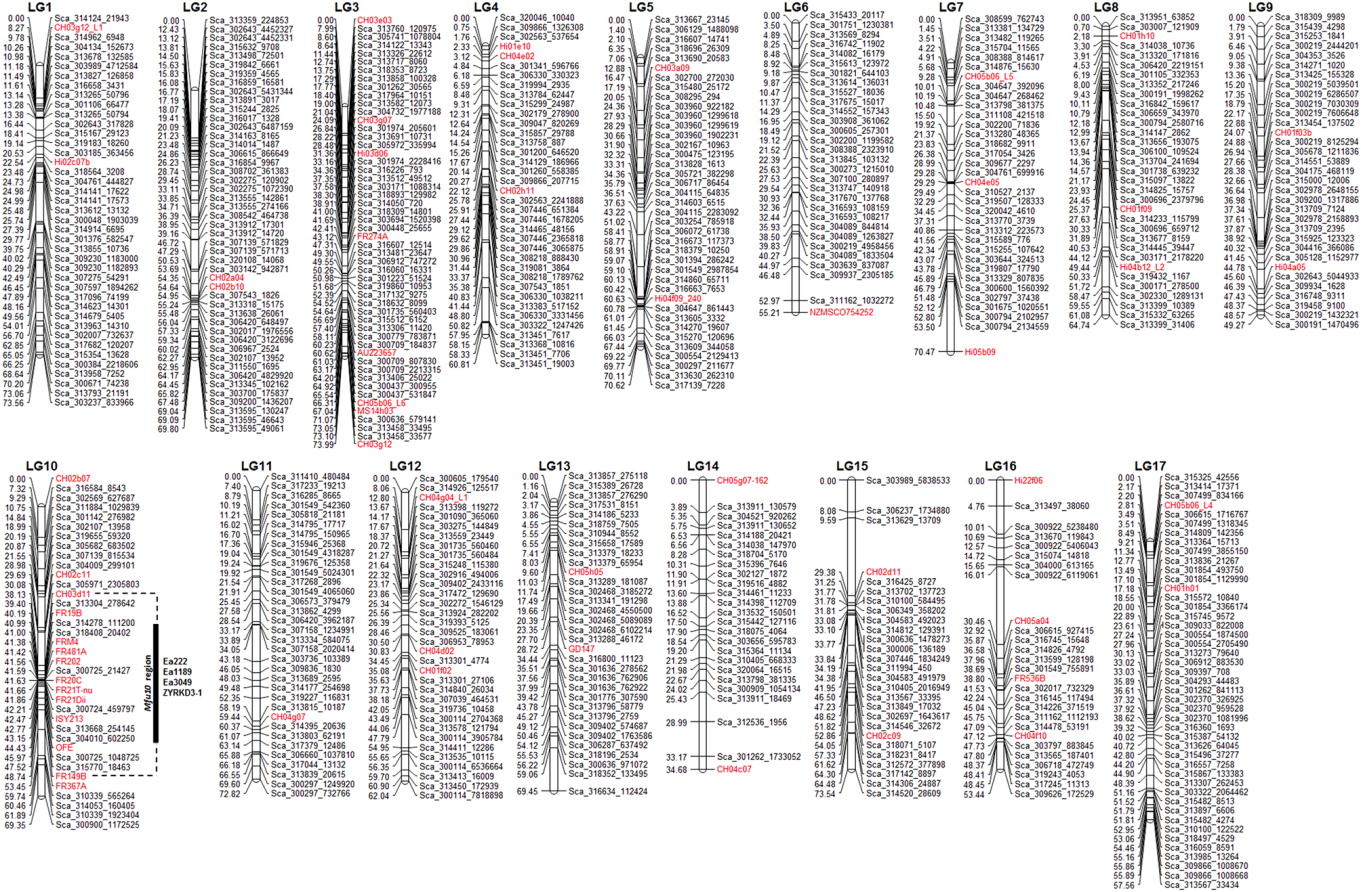
Integrated dense genetic map of MAL0045 comprising 560 tGBS SNPs and 53 SSR markers (highlighted in red). Total length is 1081.35 cM.

Analysis with JoinMap4.0^53^ was performed using the Kosambi’s function and grouping independence LOD of 12–18. The final integrated map of *M*. *fusca* is comprised of 560 tGBS SNPs and 53 previously mapped SSR markers, 613 loci in total. All markers could be assigned to the 17 linkage groups of apple, with a total genetic length of 1081.35 cM (Fig. 1, Table 1). The total length is 1479.45 cM less than the overall genetic distance with only the 2424 SNP markers. Two SSR markers were found to be multi-locus: CH03g12 mapped on LG1 and LG3 whilst CH05b06 mapped on LG3, LG7 and LG17. Figure S3 shows the comparison of the position of markers of each *M. fusca* LG to their physical position on the respective chromosome of GDDH13^10^. The alignment of the marker sequences, i.e. both the forward and the reverse primers of the SSRs in an appropriate distance, to the GDDH13 physical chromosomes revealed 116 markers for which the best alignment hit either did not match the corresponding GDDH13 chromosome or gave no hit.

The order of most markers on the 17 LGs is in agreement with their physical order on the respective chromosomes (Fig. S3). Additionally, a homoeolog to the fire blight resistant gene *Mfu10* was mapped on chromosome 10 of GDDH13 (Fig. S3). A possible inversion can be seen in between the upper part of LG5 and some possible rearrangements on LG10 and LG14 (Fig. S3). The largest physical distance in Mb for the first marker of a LG to the start of the chromosome was observed for LG15 with 9.03 Mb, for the last marker of a LG to the end of the chromosome the distance is 16.62 Mb for LG13 and the largest distance between two markers is 16.61 Mb on LG11.

### Comparative fire blight mapping using tGBS SNP map

**Figure 2:**
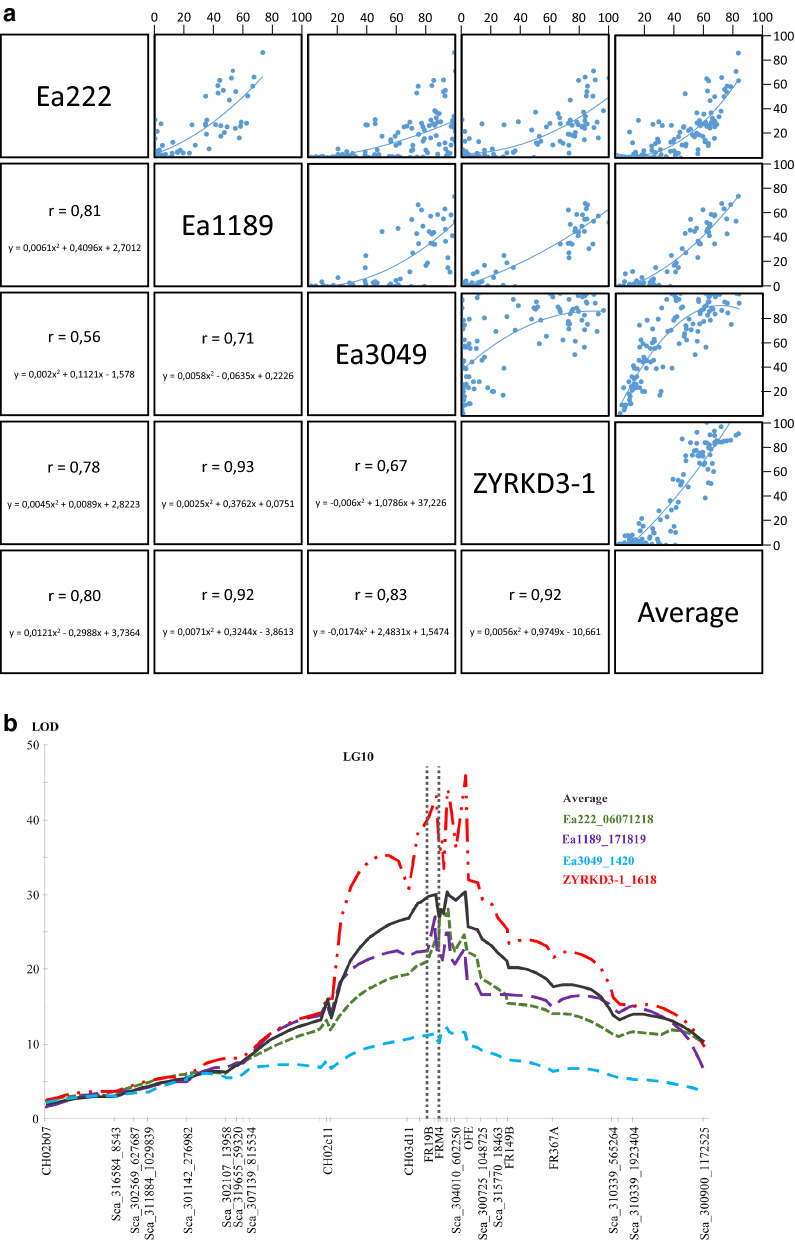
(**a**) Pairwise correlations and scatter plots for the phenotypic values of MAL0045-derived progeny. The average values for each individual for the four strains and the overall average were also used for QTL analyses. The lines represent the binominal trend line. Axes show percent lesion length (PLL). (**b**) LOD score plot of LG10 of MAL0045 for the four *E. amylovora* strains and the average value of all strains. The numbers attached after each strain name indicate the year(s) of phenotyping (e.g. Ea3049_1420 means that individuals were inoculated in 2014 and 2020).

Previously, a strong fire blight resistance QTL (*Mfu10*) was identified on LG10 of *M*. *fusca* after phenotypic evaluation of the 05210 mapping population with *Erwinia amylovora* strain Ea222^16^. *Mfu10* was found to be stable after subsequent analyses with the highly aggressive Canadian strain Ea3049^41^ as well as with a mutant strain ZYRKD3-1^42^ and its wild type strain Ea1189. Pairwise correlations and scatter plots of the four strains and the overall PLL average are presented in Fig. 2a. However, previous QTL mapping were not performed with a dense genetic map as the currently developed map. As a proof of concept, phenotypic data determined on 05210 and 09260 for the three strains and the wild type strain of ZYRKD3-1, i.e. Ea1189, were used for QTL analyses. QTL mapping for percent lesion length with Ea222, Ea3049, ZYRKD3-1, and Ea1189 as well as an average of all strains were thus performed. QTLs with significant LOD scores above the chromosome wide threshold of LOD = 3.0 were detected only on LG10 of the SNP-SSR map of *M. fusca.* Interval mapping resulted in QTLs for all *E*. *amylovora* strains and the overall average at the same position on LG10 (Fig. 2b).

Table 2 shows the markers with the highest LOD of the QTL, the position of the marker, the respective phenotypic variance explained, and the PLL of progenies with and without the resistance allele. For all strains, progenies with the least lesion were those that inherited the resistance alleles with the highest difference (65.5%) between progenies that possessed the resistance allele and those that did not observed for mutant strain ZYRKD3-1. The highest correlation of PLL between two strains was determined for ZYRKD3-1 and its wild type strain Ea1189 (Fig. 2a) with r = 0.93.

**Table 2:** Summary of QTL characteristics showing markers with the highest LOD of a QTL obtained by inoculation with different *E. amylovora* strains, the position on LG10, the LOD and the phenotypic variance explained (%), respectively.

*E. amylovora* strain	Marker with highest LOD	cM	LOD	% expl	Average PLL of progenies	
					With *R*-allele	Without *R*-allele
Ea222	FR202	41.555	28.06	62.8	3.5	32.3
Ea1189	Sca_314278_111200	40.994	27.09	87.1	4.0	47.3
ZYRKD3-1	Sca_300724_459797	42.207	44.13	82.4	9.1	74.7
Ea3049	Sca_300724_459797	42.207	12.13	37.3	46.4	81.0
Average	Sca_300724_459797	42.207	30.32	62.6	19.4	56.9

*LOD* logarithm of the odd, *R-allele* resistance allele.

### Phylogenetic tree construction with wild apples and Idared-specific SNPs

A mini diversity panel study of four wild *Malus fusca* samples (MAL0200, MAL0289, MAL0357, and MAL0768) and the two parent samples (MAL0045 and ‘Idared’) was performed with 64,664 SNPs identified for these samples. The SNPs could be genotyped in at least four of the six samples and had a minor allele frequency of ≥ 1/12 allele as well as a genotype number of ≥ 2 and an allele number equal to two. The phylogenic tree shows that all wild accessions clustered together and are much different from the domesticated parent ‘Idared’ (Fig. S4).

## Discussion

High-density marker platforms are capable of enhancing fire blight resistance breeding through fast and precise identification of fire blight QTLs^56^. In the present study, we constructed a dense genetic map of the wild apple *M*. *fusca* using tunable genotyping-by-sequencing SNP-type technology and subsequently integrated previously reported SSR data^16^ as anchor markers. Although, a high density SNP apple array, the Axiom 480 k apple array^31^, is available for apple, SNP discovery by tGBS was preferred. The array was developed to cover the genetic diversity of the cultivated apple^31^ but due to the genetic distance between *M. fusca* accessions and *M. domestica* as shown in Fig. S4, only a low percentage of apple SNPs were transferrable to *M. fusca*^16^. The thousands of SNPs detected with tGBS technology in the current study further demonstrates the high throughput and effectiveness for generating genome-wide SNP data which are suitable for genetic mapping studies^36^. The number of SNPs successfully mapped here (2424) is similar and comparable to other studies in apple e.g. 2436^33^ and 2590^57^. However, the high degree of unexplainable double recombinations found in the tGBS SNP map data (not shown) is a disadvantage and the question arise what the reason could be. This situation leads to the overestimation of the sizes of linkage groups (LGs) allowing them to be partly incomparable to reference maps in apple^19^. Besides, redundancy (no recombination of SNPs) was also high leading to clustering of scaffolds however; this is expected in a population size as in the current study. Nevertheless after the removal of such SNPs and the integration of SSR data, the resultant map (Fig. 1) spanned 1081.35 cM (1479.45 cM less than the original tGBS SNP map) (Fig. S2). The initial map reported by^16^ spanned 889 cM with markers spread across 20 linkage groups. In contrast, markers in this newly integrated map spread across the recognized 17 linkage groups of apple and is more comparable to 1140 cM and 1450 cM for apple reference maps of ‘Fiesta’ and ‘Discovery’, respectively, reported by^19^. Even after enriching the ‘Fiesta’ and ‘Discovery’ maps with more markers, Silfverberg-Dilworth et al.^20^ reported genetic lengths of 1145.3 cM and 1417.1 cM respectively for both maps. Moreover, using GBS technology, Norelli et al.^34^ reported a total genetic length ranging from 1230.2 to 1722.9 cM for a map of *M*. *sieversii* comprising GBS markers and SSRs whilst GBS markers spanned 1271 cM for a ‘Golden Delicious’ × ‘Scarlet Spur’ population^33^ and 1350.1 cM for ‘Hongro’ × ‘Alps otome’ apple population^57^. The order of the SSRs on the 17 LGs of *M. fusca* corresponds to their positions on the apple-integrated map^32^. Furthermore, the order of most markers of *M. fusca* LGs is in agreement with their physical position on the respective chromosome of GDDH13. Only few possible inversions have been observed. Nevertheless, in all LGs markers have been mapped for which no position could be mapped on the respective chromosome. This could be because only the best hit was taken into account, to typing errors in marker analysis, to errors in genetic mapping or to the genetic distance of *M. domestica* and *M. fusca* (Fig. S4). Duan et al.^58^ analysed the population structure of 117 apple cultivars and wild species accessions and constructed phylogenetic tree showing the large genetic distance of *M. domestica* and *M. fusca*. By comparing the LGs to the chromosomes, large parts from the start or the end of some chromosome end are not identified by a marker. Additionally, some large gaps in between two adjacent markers on a chromosome were identified. Large gaps are explainable by the relatively low density of markers and the smaller nuclear DNA content of *M. fusca*. The median nuclear DNA content of five *M. fusca* accessions was determined as 1.464 pg compared to the median nuclear DNA content of 1.514 pg for *M. domestica*^9^.

The *M. fusca* (MAL0045) × ‘Idared’ 05210 population was initially developed for mapping the fire blight resistance locus of this crabapple^25^. The major locus (*Mfu10*), which confers resistance to fire blight, caused by the bacterium, *Erwinia amylovora*, was detected following artificial shoot inoculation with *E*. *amylovora* strain, Ea222. Subsequent studies found *Mfu10* to be stable following phenotypic evaluation with the highly aggressive Canadian strain, Ea3049^41^ and the AvrRpt2_EA_ mutant ZYRKD3-1^42^. Ea222 and Ea3049 possess the C-allele and the S-allele, respectively, of AvrRpt2_EA_ effector of *E*. *amylovora* whereas the effector is deleted in strain ZYRKD3-1^59,60^. The findings of Vogt et al.^60^ imply that the S-allele of AvrRpt2_EA_ is responsible for aggressive virulence in *Malus* genotypes and the deletion of the effector gene (ZYRKD3-1) leads to a gene-for-gene relationship in *Malus* × *robusta* 5 (Mr5)—*E. amylovora* system. Although the highly aggressive Canadian strain does not affect MAL0045, several of its progeny individuals were highly susceptible with only a few showing strong resistance to this strain^41^, resulting in the low LOD compared to the other strains in Fig. 2b. In similar studies with Ea3049, Peil et al.^61^ and Wöhner et al.^22^ reported the breakdown of the major QTL on LG3 of Mr5 but identified minor QTLs in different linkage groups. Previous mapping studies using MAL0045-derived progeny and the three strains mentioned above did not identify any minor QTLs leading to the assumption that the lack of density of MAL0045 genetic map could be the hindrance especially as the development of an improved map of Mr5 facilitated the detection of more minor fire blight QTLs with Ea3049^22^.

Yet, QTL mapping with the dense map developed in the current study did not detect any minor locus. With the knowledge gained from Mr5 fire blight resistance and results from the current study, we therefore hypothesize that detection of minor QTLs does not only depend on a dense genetic map, but also largely depends on the *E*. *amylovora* strain. Fire blight resistance is strain dependent and in Mr5, no minor QTLs were detected with Mr5-avirulent strains^54,62–64^, but only with strains virulent to Mr5^22,61^. It is thus plausible that the failure to detect a minor locus with the strains used in the current study is down to their interactions with MAL0045 and MAL0045-derived progeny. Nevertheless, it was worthwhile to construct such a map for this highly resistant wild species accession, as future-mapping studies with different pathogen strains, including mutant strains, will then eliminate the lack of a dense genetic map. To our knowledge, this is the first report of QTL mapping in *Malus* using the average data of four different strains of *E*. *amylovora*. Further genetic studies would focus on if bacterial effector mutants will be able to overcome MAL0045 resistance and if a second resistance factor will be identified, which due to previous results^16,41,42^, must be present in this accession. A resistance not overcome by any strain of the pathogen is valuable to breeders to develop resistant cultivars.

## Supplementary information

Supplementary Figure S1.

Supplementary Figure S2.

Supplementary Figure S3.

Supplementary Figure S4.

Supplementary Table S1.

## References

[1] Viereck LA, Little Elbert LJ (2007). Alaska Trees and Shrubs.

[2] Qian G-Z, Liu L-F, Tang G-G (2006). A new selection of *Malus* (Rosaceae) from China. Ann. Bot. Fenn..

[3] Robinson J, Harris S, Juniper B (2001). Taxonomy of the genus *Malus* Mill. (*Rosaceae*) with emphasis on the cultivated apple, *Malus domestica *Borkh. Plant Syst. Evol..

[4] Fiala JL (1994). Flowering Crabapples: The Genus *Malus*.

[5] Vavilov NI (1926). Studies on the origin of cultivated plants. Bull. Appl. Bot.

[6] Zohary D, Hopf M (2000). Domestication of Plants in the Old World: The Origin and Spread of Cultivated Plants in West Asia, Europe and the Nile Valley.

[7] Cornille A, Giraud T, Smulders MJ, Roldán-Ruiz I, Gladieux P (2014). The domestication and evolutionary ecology of apples. Trends Genet..

[8] Hanke, M.-V., Flachowsky, H., Peil, A. & Emeriewen, O. F. in *Biotechnology of Fruit and Nut Crops Biotechnology in Agricultural Series* (eds R. Litz, F. Pliego-Alfaro, & J. I. Hormaza) 440–473 (CAB International, Cambridge, 2020).

[9] Höfer M, Meister A (2010). Genome size variation in *Malus* species. Journal of Botany.

[10] Daccord N (2017). High-quality de novo assembly of the apple genome and methylome dynamics of early fruit development. Nat. Genet..

[11] Velasco R (2010). The genome of the domesticated apple (*Malus × domestica* Borkh.). Nat. Genet..

[12] Conner PJ, Brown SK, Weeden NF (1997). Randomly amplified polymorphic DNA-based genetic linkage maps of three apple cultivars. J. Am. Soc. Hortic. Sci..

[13] Hemmat M, Weedon N, Manganaris A, Lawson D (1994). Molecular marker linkage map for apple. J. Hered..

[14] Maliepaard C (1998). Aligning male and female linkage maps of apple (*Malus pumila* Mill.) using multi-allelic markers. Theor. Appl. Genet..

[15] Celton JM, Tustin DS, Chagne D, Gardiner SE (2009). Construction of a dense genetic linkage map for apple rootstocks using SSRs developed from *Malus* ESTs and *Pyrus* genomic sequences. Tree Genet. Genomes.

[16] Emeriewen OF (2014). Identification of a major quantitative trait locus for resistance to fire blight in the wild apple species *Malus fusca*. Mol. Breeding.

[17] Han Y (2011). Integration of physical and genetic maps in apple confirms whole-genome and segmental duplications in the apple genome. J. Exp. Bot..

[18] Liebhard R (2002). Development and characterisation of 140 new microsatellites in apple (*Malus × domestica* Borkh.). Mol. Breeding.

[19] Liebhard R, Koller B, Gianfranceschi L, Gessler C (2003). Creating a saturated reference map for the apple (*Malus × domestica* Borkh.) genome. Theor. Appl. Genet..

[20] Silfverberg-Dilworth E (2006). Microsatellite markers spanning the apple (*Malus × domestica* Borkh.) genome. Tree Genet. Genomes.

[21] Wang A (2012). EST contig-based SSR linkage maps for *Malus × domestica* cv. Royal Gala and an apple scab resistant accession of *M. sieversii*, the progenitor species of domestic apple. Mol. Breeding.

[22] Wöhner TW (2014). QTL mapping of fire blight resistance in *Malus* x*robusta* 5 after inoculation with different strains of *Erwinia amylovora*. Mol. Breeding.

[23] Guilford P (1997). Microsatellites in *Malus* × *domestica* (apple): abundance, polymorphism and cultivar identification. Theor. Appl. Genet..

[24] Jaccoud D, Peng K, Feinstein D, Kilian A (2001). Diversity arrays: a solid state technology for sequence information independent genotyping. Nucleic Acids Res..

[25] Emeriewen O (2014). Evidence of a major QTL for fire blight resistance in the apple wild species *Malus fusca*. Acta Hortic..

[26] Sánchez-Sevilla JF (2015). Diversity Arrays Technology (DArT) marker platforms for diversity analysis and linkage mapping in a complex crop, the octoploid cultivated strawberry (*Fragaria* × *ananassa*). PLoS ONE.

[27] Schouten HJ (2012). Diversity arrays technology (DArT) markers in apple for genetic linkage maps. Mol. Breeding.

[28] Soriano JM (2009). Identification and mapping of the novel apple scab resistance gene Vd3. Tree Genet. Genomes.

[29] Chagne D (2012). Genome-wide SNP detection, validation, and development of an 8K SNP array for apple. PLoS ONE.

[30] Bianco L (2014). Development and validation of a 20K single nucleotide polymorphism (SNP) whole genome genotyping array for apple (*Malus* × *domestica* Borkh). PLoS ONE.

[31] Bianco L (2016). Development and validation of the Axiom Apple480K SNP genotyping array. Plant J..

[32] Di Pierro EA (2016). A high-density, multi-parental SNP genetic map on apple validates a new mapping approach for outcrossing species. Hortic. Res..

[33] Gardner KM (2014). Fast and cost-effective genetic mapping in apple using next-generation sequencing. G3 Genes Genomes Genet..

[34] Norelli JL (2017). Genotyping-by-sequencing markers facilitate the identification of quantitative trait loci controlling resistance to *Penicillium expansum* in *Malus sieversii*. PLoS ONE.

[35] Elshire RJ (2011). A robust, simple genotyping-by-sequencing (GBS) approach for high diversity species. PLoS ONE.

[36] Poland JA, Brown PJ, Sorrells ME, Jannink J-L (2012). Development of high-density genetic maps for barley and wheat using a novel two-enzyme genotyping-by-sequencing approach. PLoS ONE.

[37] Poland JA, Rife TW (2012). Genotyping-by-sequencing for plant breeding and genetics. Plant Genome.

[38] Kumar S (2017). Genotyping-by-sequencing of pear (*Pyrus* spp.) *a*ccessions unravels novel patterns of genetic diversity and selection footprints. Hortic. Res..

[39] Ott A (2017). tGBS genotyping-by-sequencing enables reliable genotyping of heterozygous loci. Nucleic Acids Res..

[40] Emeriewen OF (2018). Towards map-based cloning of FB_Mfu10: identification of a receptor-like kinase candidate gene underlying the *Malus fusca* fire blight resistance locus on linkage group 10. Mol. Breeding.

[41] Emeriewen OF, Richter K, Hanke MV, Malnoy M, Peil A (2015). The fire blight resistance QTL of *Malus fusca* (Mfu10) is affected but not broken down by the highly virulent Canadian *Erwinia amylovora* strain E2002A. Eur. J. Plant Pathol..

[42] Emeriewen O, Richter K, Hanke M-V, Malnoy M, Peil A (2017). Further insights into *Malus fusca* fire blight resistance. J. Plant Pathol..

[43] Chou, H., Sutton, G., Glodek, A. & Scott, J. in *Proceedings of the Tenth Annual Genome Sequencing and Annotation Conference (GSAC X).*

[44] Li S, Chou H-H (2004). LUCY2: an interactive DNA sequence quality trimming and vector removal tool. Bioinformatics.

[45] Ewing B, Green P (1998). Base-calling of automated sequencer traces using phred II. Error probabilities. Genome Res..

[46] Ewing B, Hillier L, Wendl MC, Green P (1998). Base-calling of automated sequencer traces usingPhred I. Accuracy assessment. Genome Res..

[47] Wu TD, Nacu S (2010). Fast and SNP-tolerant detection of complex variants and splicing in short reads. Bioinformatics.

[48] Li X (2012). Genic and nongenic contributions to natural variation of quantitative traits in maize. Genome Res..

[49] Li T (2018). Genetic characterization of inbred lines from Shaan A and B groups for identifying loci associated with maize grain yield. BMC Genet..

[50] Zheng Z (2018). Genetic diversity, oopulation structure, and botanical variety of 320 global peanut accessions revealed through tunable genotyping-by-sequencing. Sci. Rep..

[51] Guindon S (2010). New algorithms and methods to estimate maximum-likelihood phylogenies: assessing the performance of PhyML 3.0. Syst. Biol..

[52] Broman K, Wu H, Sen S, Churchill G (2003). R/qtl: QTL mapping in experimental crosses. Bioinformatics.

[53] Van Oojien J (2006). JoinMap 4, software for the calculation of genetic linkage maps in experimental population.

[54] Peil A (2007). Strong evidence for a fire blight resistance gene of *Malus* ×*robusta* located on linkage group 3. Plant Breeding.

[55] Van Ooijen JW (2004). MapQTL5, Software for the mapping of quantitative trait loci in experimental populations.

[56] Peil, A., Emeriewen, O. F., Khan, A., Kostick, S. & Malnoy, M. Status of fire blight resistance breeding in Malus.

[57] Ban SH, Choi C (2018). Development of an apple F1 segregating population genetic linkage map using genotyping-by-sequencing. Plant Breeding Biotechnol..

[58] Duan N (2017). Genome re-sequencing reveals the history of apple and supports a two-stage model for fruit enlargement. Nat. Commun..

[59] Emeriewen OF, Wöhner T, Flachowsky H, Peil A (2019). Malus Hosts-Erwinia amylovora interactions: strain pathogenicity and resistance mechanisms. Front. Plant Sci..

[60] Vogt I (2013). Gene-for-gene relationship in the host-pathogen system *Malus* x*robusta* 5-*Erwinia amylovora*. New Phytol..

[61] Peil A, Flachowsky H, Hanke M-V, Richter K, Rode J (2011). Inoculation of *Malus* ×*robusta* 5 progeny with a strain breaking resistance to fire blight reveals a minor QTL on LG5. Acta Hortic..

[62] Gardiner SE (2012). Putative resistance gene markers associated with quantitative trait loci for fire blight resistance in *Malus* '*Robusta* 5' accessions. BMC Genet..

[63] Peil A (2008). Confirmation of the fire blight QTL of *Malus* × *robusta* 5 on linkage group 3. Acta Hortic..

[64] Peil A (2019). Mapping of fire blight resistance in *Malus × robusta *5 flowers following artificial inoculation. BMC Plant Biol..

