# Supplementary material for: Construction of a dense genetic map of the Malus fusca fire blight resistant accession MAL0045 using tunable genotyping-by-sequencing SNPs and microsatellites
Source: Sci Rep. 2020 Oct 1;10:16358. doi: 10.1038/s41598-020-73393-6 (PMC7529804; doi:10.1038/s41598-020-73393-6)
Supplement: Supplementary file 1 — Supplementary Figure S1. [file 41598_2020_73393_MOESM1_ESM.pdf]

Construction of a dense genetic map of the *Malus fusca* fire blight resistant accession MAL0045 using tunable genotyping-by-sequencing SNPs and microsatellites

Ofere Francis Emeriewen[1], Klaus Richter[2], Thomas Berner[3], Jens Keilwagen[3], Patrick S. Schnable[4], Mickael Malnoy[5] and Andreas Peil[1]

[1]Julius Kühn-Institut (JKI), Federal Research Centre for Cultivated Plants, Institute for Breeding Research on Fruit Crops, Pillnitzer Platz 3a, 01326 Dresden, Germany

[2]Julius Kühn-Institut (JKI), Federal Research Centre for Cultivated Plants, Institute for Resistance Research and Stress Tolerance, Erwin-Baur-Str. 27, 06484, Quedlinburg, Germany

[3]Julius Kühn-Institut (JKI), Federal Research Centre for Cultivated Plants, Institute for Biosafety in Plant Biotechnology, Erwin-Baur-Str. 27, 06484, Quedlinburg, Germany

[4]Data2Bio LLC, Ames, IA 50011-3650, USA

[5]Research and Innovation Centre, Genomics and Biology of Fruit Crops Department, Fondazione Edmund Mach, Via E. Mach, 1 – 38010 San Michele all 'Adige (Trentino) Italy

Corresponding authors: Ofere Francis Emeriewen, Andreas Peil

Julius Kühn-Institut (JKI)
Federal Research Centre for Cultivated Plants
Institute for Breeding Research on Fruit Crops
Pillnitzer Platz 3a
01326 Dresden
Germany

ofere.emeriewen@julius-kuehn.de
andreas.peil@julius-kuehn.de

Figure S1. Mapping population SNP classification

MAL0045 / IDARED	AA	a	BB
AA	**X**	**1:1**	**X**
AB	**1:1**	**1:2:1**	**1:1**
BB	**X**	**1:1**	**X**
